# Supplementary material for: Immune Pressure on Polymorphous Influenza B Populations Results in Diverse Hemagglutinin Escape Mutants and Lineage Switching
Source: Vaccines (Basel). 2020 Mar 11;8(1):125. doi: 10.3390/vaccines8010125 (PMC7157493; doi:10.3390/vaccines8010125)
Supplement: Supplementary file 1 [file vaccines-08-00125-s001.pdf]

Supplementary File S1

NGS Sequence Data for Polymorphous Influenza B Populations

RNA was extracted from 14 viruses and used to make DNA libraries which were sequenced. Two extractions were performed for each virus and two reads were performed for each library. The first RNA extraction resulted in higher quality RNA and the subsequent sequence reads from this were used in the analysis. The sequence ID for each is listed in the table below. The raw sequence data is available from the HIVE database: https://hive.biochemistry.gwu.edu/review/Immune%20pressure%20on%20polymorphous%20influenza%20B%20populations%20results%20in%20diverse%20escape%20mutants%20and%20lineage%20switching

Data for each read includes a unique ID, file names, and the number of sequence records is displayed in the upper panel. The file names include the virus strain (CO for B/Colorado/06/2017 or PK for B/Phuket/3073/2013) and the sera used (F09, M50, M74, M09, M56 and F16), with upper or lower case used to separate the two extractions. Virus passage without sera is labeled WT (wildtype).

The sequence data for each read can be accessed using the lower panel. When the sequences tab is selected the user can chose the number of sequences to be displayed. Additional information (histograms, taxonomy etc.) can be viewed by selecting the appropriate tabs.

Virus	Sera	Extraction	Read	ID
B/Colorado/06/2017	None (WT)	1	1	586421
B/Colorado/06/2017	None (WT)	1	2	586420
B/Colorado/06/2017	None (WT)	2	1	586393
B/Colorado/06/2017	None (WT)	2	2	586392
B/Colorado/06/2017	f09	1	1	586391
B/Colorado/06/2017	f09	1	2	586390
B/Colorado/06/2017	F09	2	1	586419
B/Colorado/06/2017	F09	2	2	586418
B/Colorado/06/2017	m50	1	1	586413
B/Colorado/06/2017	m50	1	2	586412
B/Colorado/06/2017	M50	2	1	586385
B/Colorado/06/2017	M50	2	2	586384
B/Colorado/06/2017	m74	1	1	586409
B/Colorado/06/2017	m74	1	2	586408
B/Colorado/06/2017	M74	2	1	586381
B/Colorado/06/2017	M74	2	2	586380
B/Colorado/06/2017	m09	1	1	586415
B/Colorado/06/2017	m09	1	2	586414
B/Colorado/06/2017	M09	2	1	586387
B/Colorado/06/2017	M09	2	2	586386
B/Colorado/06/2017	m56	1	1	586411
B/Colorado/06/2017	m56	1	2	586410
B/Colorado/06/2017	M56	2	1	586383
B/Colorado/06/2017	M56	2	2	586382
B/Colorado/06/2017	f16	1	1	586417
B/Colorado/06/2017	f16	1	2	586416
B/Colorado/06/2017	F16	2	1	586389
B/Colorado/06/2017	F16	2	2	586388
BPhuket/3073/2013	None (WT)	1	1	586407
BPhuket/3073/2013	None (WT)	1	2	586406
BPhuket/3073/2013	None (WT)	2	1	586379
BPhuket/3073/2013	None (WT)	2	2	586378

BPhuket/3073/2013	f09	1	1	586405
BPhuket/3073/2013	f09	1	2	586404
BPhuket/3073/2013	F09	2	1	586377
BPhuket/3073/2013	F09	2	2	586376
BPhuket/3073/2013	m50	1	1	586399
BPhuket/3073/2013	m50	1	2	586398
BPhuket/3073/2013	M50	2	1	586371
BPhuket/3073/2013	M50	2	2	586370
BPhuket/3073/2013	m74	1	1	586395
BPhuket/3073/2013	m74	1	2	586394
BPhuket/3073/2013	M74	2	1	586367
BPhuket/3073/2013	M74	2	2	586366
BPhuket/3073/2013	m09	1	1	586401
BPhuket/3073/2013	m09	1	2	586400
BPhuket/3073/2013	M09	2	1	586373
BPhuket/3073/2013	M09	2	2	586372
BPhuket/3073/2013	m56	1	1	586397
BPhuket/3073/2013	m56	1	2	586396
BPhuket/3073/2013	M56	2	1	586369
BPhuket/3073/2013	M56	2	2	586368
BPhuket/3073/2013	f16	1	1	586403
BPhuket/3073/2013	f16	1	2	586402
BPhuket/3073/2013	F16	2	1	586375
BPhuket/3073/2013	F16	2	2	586374
